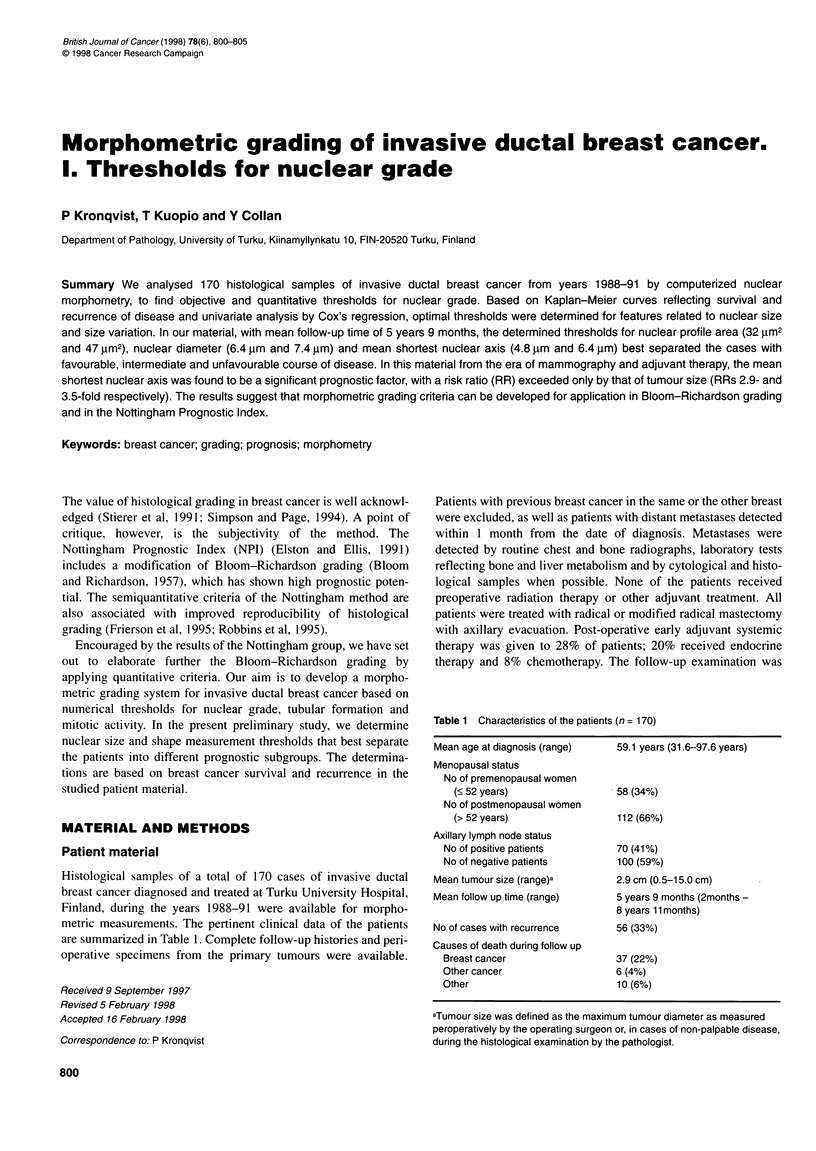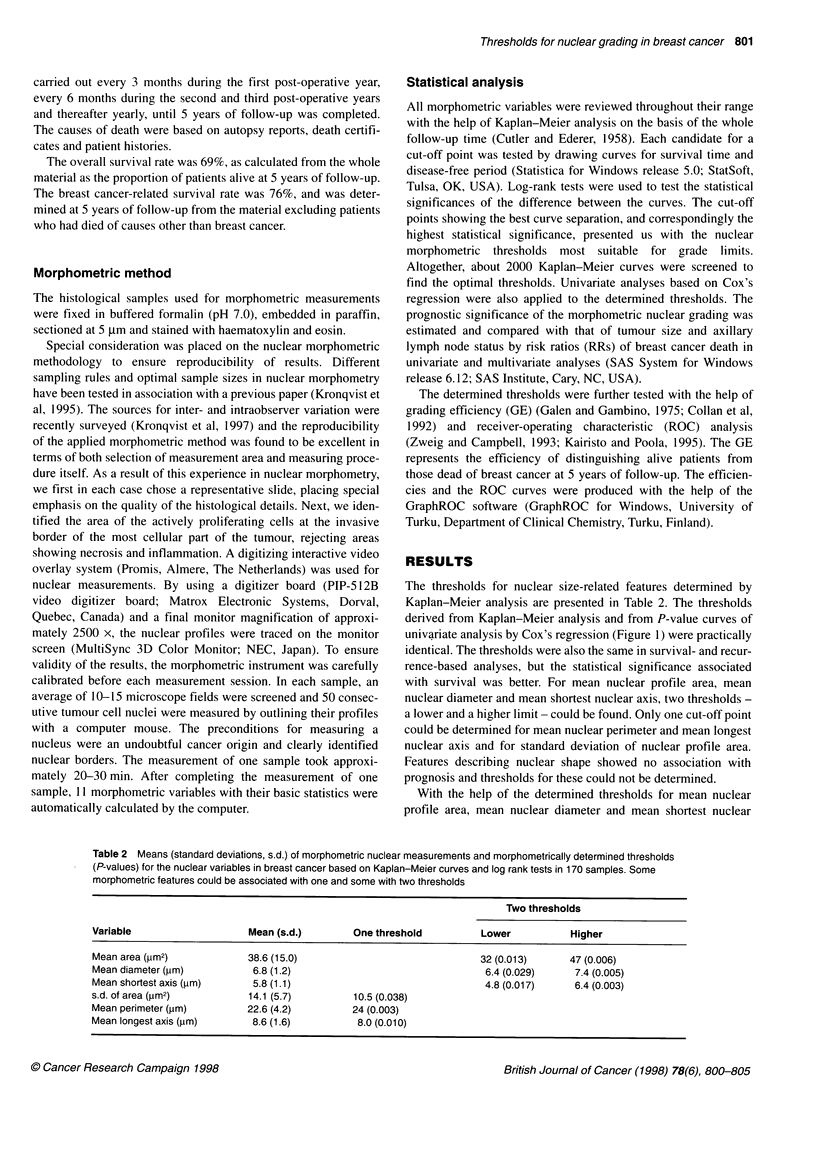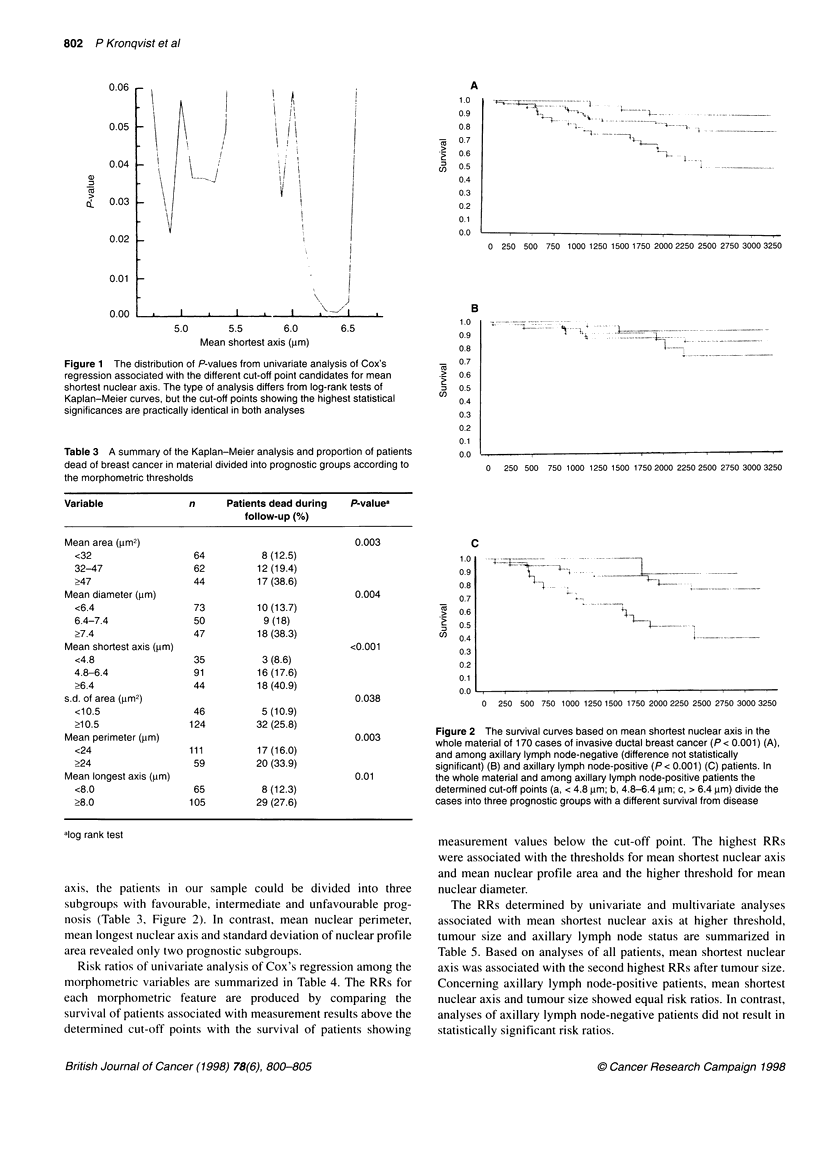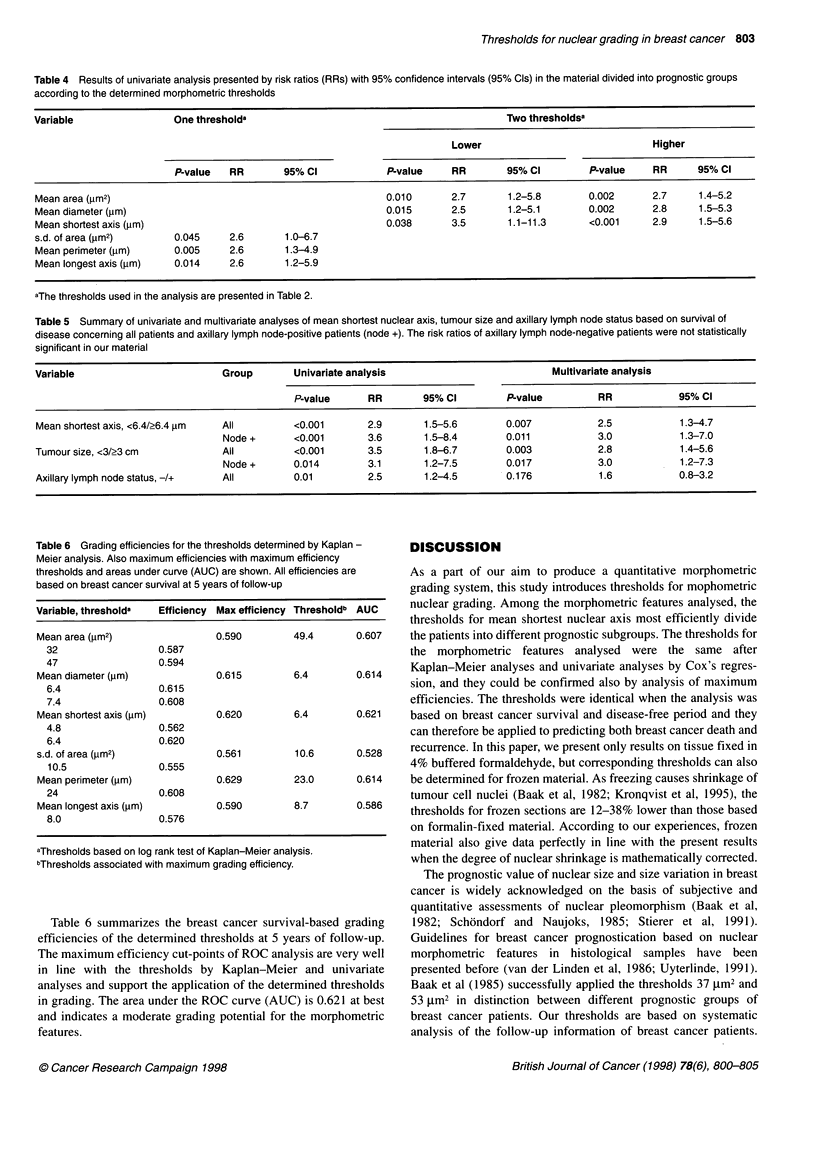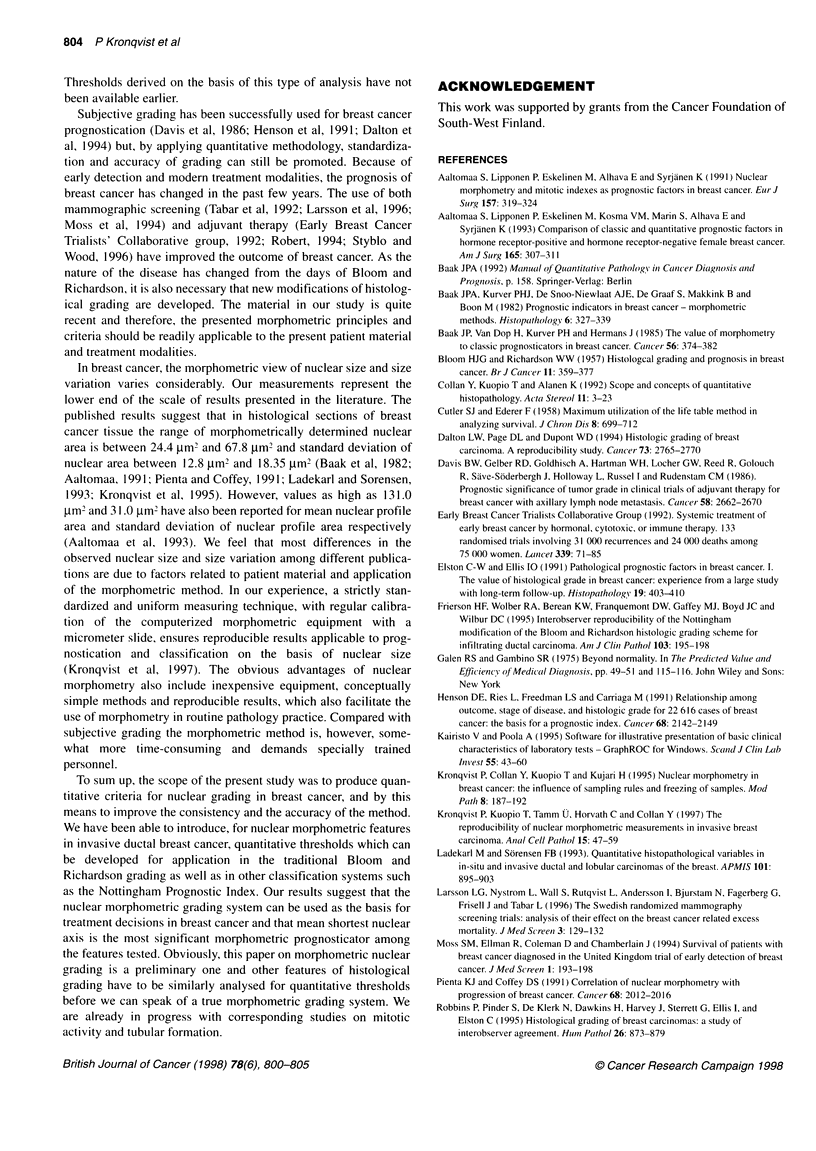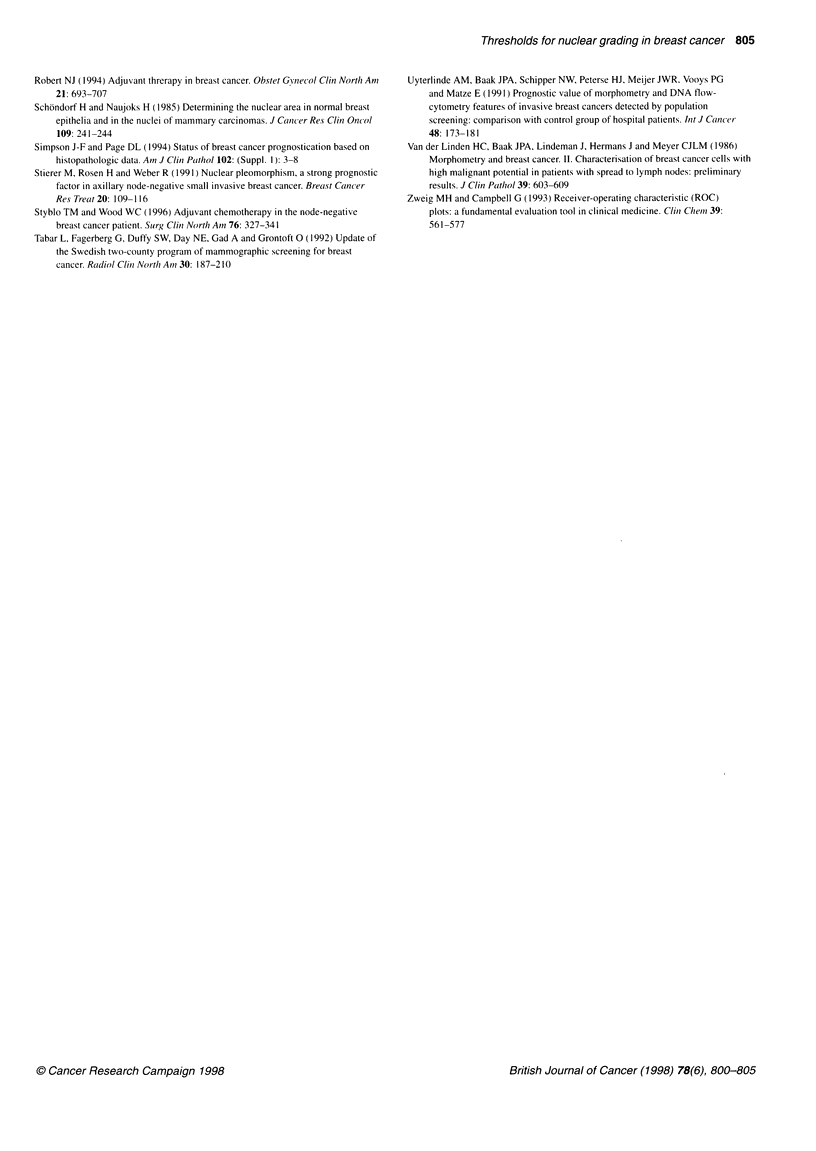# Morphometric grading of invasive ductal breast cancer. I. Thresholds for nuclear grade.

**DOI:** 10.1038/bjc.1998.582

**Published:** 1998-09

**Authors:** P. Kronqvist, T. Kuopio, Y. Collan

**Affiliations:** Department of Pathology, University of Turku, Finland.

## Abstract

We analysed 170 histological samples of invasive ductal breast cancer from years 1988-91 by computerized nuclear morphometry, to find objective and quantitative thresholds for nuclear grade. Based on Kaplan-Meier curves reflecting survival and recurrence of disease and univariate analysis by Cox's regression, optimal thresholds were determined for features related to nuclear size and size variation. In our material, with mean follow-up time of 5 years 9 months, the determined thresholds for nuclear profile area (32 microm2 and 47 microm2), nuclear diameter (6.4 microm and 7.4 microm) and mean shortest nuclear axis (4.8 microm and 6.4 microm) best separated the cases with favourable, intermediate and unfavourable course of disease. In this material from the era of mammography and adjuvant therapy, the mean shortest nuclear axis was found to be a significant prognostic factor, with a risk ratio (RR) exceeded only by that of tumour size (RRs 2.9- and 3.5-fold respectively). The results suggest that morphometric grading criteria can be developed for application in Bloom-Richardson grading and in the Nottingham Prognostic Index.


British Journal of Cancer (1998) 78(6), 800–805
© 1998 Cancer Research Campaign

### Morphometric grading of invasive ductal breast cancer. I. Thresholds for nuclear grade

**P Kronqvist, T Kuopio and Y Collan**

Department of Pathology, University of Turku, Kiinamyllynkatu 10, FIN-20520 Turku, Finland

**Summary** We analysed 170 histological samples of invasive ductal breast cancer from years 1988–91 by computerized nuclear morphometry, to find objective and quantitative thresholds for nuclear grade. Based on Kaplan–Meier curves reflecting survival and recurrence of disease and univariate analysis by Cox's regression, optimal thresholds were determined for features related to nuclear size and size variation. In our material, with mean follow-up time of 5 years 9 months, the determined thresholds for nuclear profile area (32 $\mu m^2$ and 47 $\mu m^2$), nuclear diameter (6.4 μm and 7.4 μm) and mean shortest nuclear axis (4.8 μm and 6.4 μm) best separated the cases with favourable, intermediate and unfavourable course of disease. In this material from the era of mammography and adjuvant therapy, the mean shortest nuclear axis was found to be a significant prognostic factor, with a risk ratio (RR) exceeded only by that of tumour size (RRs 2.9- and 3.5-fold respectively). The results suggest that morphometric grading criteria can be developed for application in Bloom–Richardson grading and in the Nottingham Prognostic Index.

**Keywords:** breast cancer; grading; prognosis; morphometry

The value of histological grading in breast cancer is well acknowledged (Stierer et al, 1991; Simpson and Page, 1994). A point of critique, however, is the subjectivity of the method. The Nottingham Prognostic Index (NPI) (Elston and Ellis, 1991) includes a modification of Bloom–Richardson grading (Bloom and Richardson, 1957), which has shown high prognostic potential. The semiquantitative criteria of the Nottingham method are also associated with improved reproducibility of histological grading (Frierson et al, 1995; Robbins et al, 1995).

Encouraged by the results of the Nottingham group, we have set out to elaborate further the Bloom–Richardson grading by applying quantitative criteria. Our aim is to develop a morphometric grading system for invasive ductal breast cancer based on numerical thresholds for nuclear grade, tubular formation and mitotic activity. In the present preliminary study, we determine nuclear size and shape measurement thresholds that best separate the patients into different prognostic subgroups. The determinations are based on breast cancer survival and recurrence in the studied patient material.

### MATERIAL AND METHODS

### Patient material

Histological samples of a total of 170 cases of invasive ductal breast cancer diagnosed and treated at Turku University Hospital, Finland, during the years 1988–91 were available for morphometric measurements. The pertinent clinical data of the patients are summarized in Table 1. Complete follow-up histories and peri-operative specimens from the primary tumours were available. Patients with previous breast cancer in the same or the other breast were excluded, as well as patients with distant metastases detected within 1 month from the date of diagnosis. Metastases were detected by routine chest and bone radiographs, laboratory tests reflecting bone and liver metabolism and by cytological and histological samples when possible. None of the patients received preoperative radiation therapy or other adjuvant treatment. All patients were treated with radical or modified radical mastectomy with axillary evacuation. Post-operative early adjuvant systemic therapy was given to 28% of patients; 20% received endocrine therapy and 8% chemotherapy. The follow-up examination was

*Received 9 September 1997*
*Revised 5 February 1998*
*Accepted 16 February 1998*

*Correspondence to:* P Kronqvist

**Table 1** Characteristics of the patients ($n$ = 170)

| | |
|---|---|
| Mean age at diagnosis (range) | 59.1 years (31.6–97.6 years) |
| Menopausal status | |
| No of premenopausal women (≤ 52 years) | 58 (34%) |
| No of postmenopausal women (> 52 years) | 112 (66%) |
| Axillary lymph node status | |
| No of positive patients | 70 (41%) |
| No of negative patients | 100 (59%) |
| Mean tumour size (range)[a] | 2.9 cm (0.5–15.0 cm) |
| Mean follow up time (range) | 5 years 9 months (2months – 8 years 11months) |
| No of cases with recurrence | 56 (33%) |
| Causes of death during follow up | |
| Breast cancer | 37 (22%) |
| Other cancer | 6 (4%) |
| Other | 10 (6%) |

[a]Tumour size was defined as the maximum tumour diameter as measured peroperatively by the operating surgeon or, in cases of non-palpable disease, during the histological examination by the pathologist.

carried out every 3 months during the first post-operative year, every 6 months during the second and third post-operative years and thereafter yearly, until 5 years of follow-up was completed. The causes of death were based on autopsy reports, death certificates and patient histories.

The overall survival rate was 69%, as calculated from the whole material as the proportion of patients alive at 5 years of follow-up. The breast cancer-related survival rate was 76%, and was determined at 5 years of follow-up from the material excluding patients who had died of causes other than breast cancer.

### Morphometric method

The histological samples used for morphometric measurements were fixed in buffered formalin (pH 7.0), embedded in paraffin, sectioned at 5 μm and stained with haematoxylin and eosin.

Special consideration was placed on the nuclear morphometric methodology to ensure reproducibility of results. Different sampling rules and optimal sample sizes in nuclear morphometry have been tested in association with a previous paper (Kronqvist et al, 1995). The sources for inter- and intraobserver variation were recently surveyed (Kronqvist et al, 1997) and the reproducibility of the applied morphometric method was found to be excellent in terms of both selection of measurement area and measuring procedure itself. As a result of this experience in nuclear morphometry, we first in each case chose a representative slide, placing special emphasis on the quality of the histological details. Next, we identified the area of the actively proliferating cells at the invasive border of the most cellular part of the tumour, rejecting areas showing necrosis and inflammation. A digitizing interactive video overlay system (Promis, Almere, The Netherlands) was used for nuclear measurements. By using a digitizer board (PIP-512B video digitizer board; Matrox Electronic Systems, Dorval, Quebec, Canada) and a final monitor magnification of approximately 2500 ×, the nuclear profiles were traced on the monitor screen (MultiSync 3D Color Monitor; NEC, Japan). To ensure validity of the results, the morphometric instrument was carefully calibrated before each measurement session. In each sample, an average of 10–15 microscope fields were screened and 50 consecutive tumour cell nuclei were measured by outlining their profiles with a computer mouse. The preconditions for measuring a nucleus were an undoubtful cancer origin and clearly identified nuclear borders. The measurement of one sample took approximately 20–30 min. After completing the measurement of one sample, 11 morphometric variables with their basic statistics were automatically calculated by the computer.

### Statistical analysis

All morphometric variables were reviewed throughout their range with the help of Kaplan–Meier analysis on the basis of the whole follow-up time (Cutler and Ederer, 1958). Each candidate for a cut-off point was tested by drawing curves for survival time and disease-free period (Statistica for Windows release 5.0; StatSoft, Tulsa, OK, USA). Log-rank tests were used to test the statistical significances of the difference between the curves. The cut-off points showing the best curve separation, and correspondingly the highest statistical significance, presented us with the nuclear morphometric thresholds most suitable for grade limits. Altogether, about 2000 Kaplan–Meier curves were screened to find the optimal thresholds. Univariate analyses based on Cox's regression were also applied to the determined thresholds. The prognostic significance of the morphometric nuclear grading was estimated and compared with that of tumour size and axillary lymph node status by risk ratios (RRs) of breast cancer death in univariate and multivariate analyses (SAS System for Windows release 6.12; SAS Institute, Cary, NC, USA).

The determined thresholds were further tested with the help of grading efficiency (GE) (Galen and Gambino, 1975; Collan et al, 1992) and receiver-operating characteristic (ROC) analysis (Zweig and Campbell, 1993; Kairisto and Poola, 1995). The GE represents the efficiency of distinguishing alive patients from those dead of breast cancer at 5 years of follow-up. The efficiencies and the ROC curves were produced with the help of the GraphROC software (GraphROC for Windows, University of Turku, Department of Clinical Chemistry, Turku, Finland).

### RESULTS

The thresholds for nuclear size-related features determined by Kaplan–Meier analysis are presented in Table 2. The thresholds derived from Kaplan–Meier analysis and from *P*-value curves of univariate analysis by Cox's regression (Figure 1) were practically identical. The thresholds were also the same in survival- and recurrence-based analyses, but the statistical significance associated with survival was better. For mean nuclear profile area, mean nuclear diameter and mean shortest nuclear axis, two thresholds – a lower and a higher limit – could be found. Only one cut-off point could be determined for mean nuclear perimeter and mean longest nuclear axis and for standard deviation of nuclear profile area. Features describing nuclear shape showed no association with prognosis and thresholds for these could not be determined.

With the help of the determined thresholds for mean nuclear profile area, mean nuclear diameter and mean shortest nuclear

**Table 2** Means (standard deviations, s.d.) of morphometric nuclear measurements and morphometrically determined thresholds (*P*-values) for the nuclear variables in breast cancer based on Kaplan–Meier curves and log rank tests in 170 samples. Some morphometric features could be associated with one and some with two thresholds

| | | | Two thresholds | |
|---|---|---|---|---|
| **Variable** | **Mean (s.d.)** | **One threshold** | **Lower** | **Higher** |
| Mean area (μm²) | 38.6 (15.0) | | 32 (0.013) | 47 (0.006) |
| Mean diameter (μm) | 6.8 (1.2) | | 6.4 (0.029) | 7.4 (0.005) |
| Mean shortest axis (μm) | 5.8 (1.1) | | 4.8 (0.017) | 6.4 (0.003) |
| s.d. of area (μm²) | 14.1 (5.7) | 10.5 (0.038) | | |
| Mean perimeter (μm) | 22.6 (4.2) | 24 (0.003) | | |
| Mean longest axis (μm) | 8.6 (1.6) | 8.0 (0.010) | | |

**Figure 1** The distribution of *P*-values from univariate analysis of Cox's regression associated with the different cut-off point candidates for mean shortest nuclear axis. The type of analysis differs from log-rank tests of Kaplan–Meier curves, but the cut-off points showing the highest statistical significances are practically identical in both analyses

**Table 3** A summary of the Kaplan–Meier analysis and proportion of patients dead of breast cancer in material divided into prognostic groups according to the morphometric thresholds

| Variable | *n* | Patients dead during follow-up (%) | *P*-value[a] |
|---|---|---|---|
| Mean area (μm²) | | | 0.003 |
| <32 | 64 | 8 (12.5) | |
| 32–47 | 62 | 12 (19.4) | |
| ≥47 | 44 | 17 (38.6) | |
| Mean diameter (μm) | | | 0.004 |
| <6.4 | 73 | 10 (13.7) | |
| 6.4–7.4 | 50 | 9 (18) | |
| ≥7.4 | 47 | 18 (38.3) | |
| Mean shortest axis (μm) | | | <0.001 |
| <4.8 | 35 | 3 (8.6) | |
| 4.8–6.4 | 91 | 16 (17.6) | |
| ≥6.4 | 44 | 18 (40.9) | |
| s.d. of area (μm²) | | | 0.038 |
| <10.5 | 46 | 5 (10.9) | |
| ≥10.5 | 124 | 32 (25.8) | |
| Mean perimeter (μm) | | | 0.003 |
| <24 | 111 | 17 (16.0) | |
| ≥24 | 59 | 20 (33.9) | |
| Mean longest axis (μm) | | | 0.01 |
| <8.0 | 65 | 8 (12.3) | |
| ≥8.0 | 105 | 29 (27.6) | |

[a]log rank test

**Figure 2** The survival curves based on mean shortest nuclear axis in the whole material of 170 cases of invasive ductal breast cancer ($P < 0.001$) (A), and among axillary lymph node-negative (difference not statistically significant) (B) and axillary lymph node-positive ($P < 0.001$) (C) patients. In the whole material and among axillary lymph node-positive patients the determined cut-off points (a, < 4.8 μm; b, 4.8–6.4 μm; c, > 6.4 μm) divide the cases into three prognostic groups with a different survival from disease axis, the patients in our sample could be divided into three subgroups with favourable, intermediate and unfavourable prognosis (Table 3, Figure 2). In contrast, mean nuclear perimeter, mean longest nuclear axis and standard deviation of nuclear profile area revealed only two prognostic subgroups.

Risk ratios of univariate analysis of Cox's regression among the morphometric variables are summarized in Table 4. The RRs for each morphometric feature are produced by comparing the survival of patients associated with measurement results above the determined cut-off points with the survival of patients showing measurement values below the cut-off point. The highest RRs were associated with the thresholds for mean shortest nuclear axis and mean nuclear profile area and the higher threshold for mean nuclear diameter.

The RRs determined by univariate and multivariate analyses associated with mean shortest nuclear axis at higher threshold, tumour size and axillary lymph node status are summarized in Table 5. Based on analyses of all patients, mean shortest nuclear axis was associated with the second highest RRs after tumour size. Concerning axillary lymph node-positive patients, mean shortest nuclear axis and tumour size showed equal risk ratios. In contrast, analyses of axillary lymph node-negative patients did not result in statistically significant risk ratios.

© Cancer Research Campaign 1998

**Table 4** Results of univariate analysis presented by risk ratios (RRs) with 95% confidence intervals (95% CIs) in the material divided into prognostic groups according to the determined morphometric thresholds

| Variable | One threshold[a] | | | Two thresholds[a] | | | | | |
|---|---|---|---|---|---|---|---|---|---|
| | | | | Lower | | | Higher | | |
| | *P*-value | RR | 95% CI | *P*-value | RR | 95% CI | *P*-value | RR | 95% CI |
| Mean area (μm²) | | | | 0.010 | 2.7 | 1.2–5.8 | 0.002 | 2.7 | 1.4–5.2 |
| Mean diameter (μm) | | | | 0.015 | 2.5 | 1.2–5.1 | 0.002 | 2.8 | 1.5–5.3 |
| Mean shortest axis (μm) | | | | 0.038 | 3.5 | 1.1–11.3 | <0.001 | 2.9 | 1.5–5.6 |
| s.d. of area (μm²) | 0.045 | 2.6 | 1.0–6.7 | | | | | | |
| Mean perimeter (μm) | 0.005 | 2.6 | 1.3–4.9 | | | | | | |
| Mean longest axis (μm) | 0.014 | 2.6 | 1.2–5.9 | | | | | | |

[a]The thresholds used in the analysis are presented in Table 2.

**Table 5** Summary of univariate and multivariate analyses of mean shortest nuclear axis, tumour size and axillary lymph node status based on survival of disease concerning all patients and axillary lymph node-positive patients (node +). The risk ratios of axillary lymph node-negative patients were not statistically significant in our material

| Variable | Group | Univariate analysis | | | Multivariate analysis | | |
|---|---|---|---|---|---|---|---|
| | | *P*-value | RR | 95% CI | *P*-value | RR | 95% CI |
| Mean shortest axis, <6.4/≥6.4 μm | All | <0.001 | 2.9 | 1.5–5.6 | 0.007 | 2.5 | 1.3–4.7 |
| | Node + | <0.001 | 3.6 | 1.5–8.4 | 0.011 | 3.0 | 1.3–7.0 |
| Tumour size, <3/≥3 cm | All | <0.001 | 3.5 | 1.8–6.7 | 0.003 | 2.8 | 1.4–5.6 |
| | Node + | 0.014 | 3.1 | 1.2–7.5 | 0.017 | 3.0 | 1.2–7.3 |
| Axillary lymph node status, –/+ | All | 0.01 | 2.5 | 1.2–4.5 | 0.176 | 1.6 | 0.8–3.2 |

**Table 6** Grading efficiencies for the thresholds determined by Kaplan – Meier analysis. Also maximum efficiencies with maximum efficiency thresholds and areas under curve (AUC) are shown. All efficiencies are based on breast cancer survival at 5 years of follow-up

| Variable, threshold[a] | Efficiency | Max efficiency | Threshold[b] | AUC |
|---|---|---|---|---|
| Mean area (μm²) | | 0.590 | 49.4 | 0.607 |
| 32 | 0.587 | | | |
| 47 | 0.594 | | | |
| Mean diameter (μm) | | 0.615 | 6.4 | 0.614 |
| 6.4 | 0.615 | | | |
| 7.4 | 0.608 | | | |
| Mean shortest axis (μm) | | 0.620 | 6.4 | 0.621 |
| 4.8 | 0.562 | | | |
| 6.4 | 0.620 | | | |
| s.d. of area (μm²) | | 0.561 | 10.6 | 0.528 |
| 10.5 | 0.555 | | | |
| Mean perimeter (μm) | | 0.629 | 23.0 | 0.614 |
| 24 | 0.608 | | | |
| Mean longest axis (μm) | | 0.590 | 8.7 | 0.586 |
| 8.0 | 0.576 | | | |

[a]Thresholds based on log rank test of Kaplan–Meier analysis.
[b]Thresholds associated with maximum grading efficiency.

Table 6 summarizes the breast cancer survival-based grading efficiencies of the determined thresholds at 5 years of follow-up. The maximum efficiency cut-points of ROC analysis are very well in line with the thresholds by Kaplan–Meier and univariate analyses and support the application of the determined thresholds in grading. The area under the ROC curve (AUC) is 0.621 at best and indicates a moderate grading potential for the morphometric features.

### DISCUSSION

As a part of our aim to produce a quantitative morphometric grading system, this study introduces thresholds for mophometric nuclear grading. Among the morphometric features analysed, the thresholds for mean shortest nuclear axis most efficiently divide the patients into different prognostic subgroups. The thresholds for the morphometric features analysed were the same after Kaplan–Meier analyses and univariate analyses by Cox's regression, and they could be confirmed also by analysis of maximum efficiencies. The thresholds were identical when the analysis was based on breast cancer survival and disease-free period and they can therefore be applied to predicting both breast cancer death and recurrence. In this paper, we present only results on tissue fixed in 4% buffered formaldehyde, but corresponding thresholds can also be determined for frozen material. As freezing causes shrinkage of tumour cell nuclei (Baak et al, 1982; Kronqvist et al, 1995), the thresholds for frozen sections are 12–38% lower than those based on formalin-fixed material. According to our experiences, frozen material also give data perfectly in line with the present results when the degree of nuclear shrinkage is mathematically corrected.

The prognostic value of nuclear size and size variation in breast cancer is widely acknowledged on the basis of subjective and quantitative assessments of nuclear pleomorphism (Baak et al, 1982; Schöndorf and Naujoks, 1985; Stierer et al, 1991). Guidelines for breast cancer prognostication based on nuclear morphometric features in histological samples have been presented before (van der Linden et al, 1986; Uyterlinde, 1991). Baak et al (1985) successfully applied the thresholds 37 μm² and 53 μm² in distinction between different prognostic groups of breast cancer patients. Our thresholds are based on systematic analysis of the follow-up information of breast cancer patients.

Thresholds derived on the basis of this type of analysis have not been available earlier.

Subjective grading has been successfully used for breast cancer prognostication (Davis et al, 1986; Henson et al, 1991; Dalton et al, 1994) but, by applying quantitative methodology, standardization and accuracy of grading can still be promoted. Because of early detection and modern treatment modalities, the prognosis of breast cancer has changed in the past few years. The use of both mammographic screening (Tabar et al, 1992; Larsson et al, 1996; Moss et al, 1994) and adjuvant therapy (Early Breast Cancer Trialists' Collaborative group, 1992; Robert, 1994; Styblo and Wood, 1996) have improved the outcome of breast cancer. As the nature of the disease has changed from the days of Bloom and Richardson, it is also necessary that new modifications of histological grading are developed. The material in our study is quite recent and therefore, the presented morphometric principles and criteria should be readily applicable to the present patient material and treatment modalities.

In breast cancer, the morphometric view of nuclear size and size variation varies considerably. Our measurements represent the lower end of the scale of results presented in the literature. The published results suggest that in histological sections of breast cancer tissue the range of morphometrically determined nuclear area is between 24.4 $\mu m^2$ and 67.8 $\mu m^2$ and standard deviation of nuclear area between 12.8 $\mu m^2$ and 18.35 $\mu m^2$ (Baak et al, 1982; Aaltomaa, 1991; Pienta and Coffey, 1991; Ladekarl and Sorensen, 1993; Kronqvist et al, 1995). However, values as high as 131.0 $\mu m^2$ and 31.0 $\mu m^2$ have also been reported for mean nuclear profile area and standard deviation of nuclear profile area respectively (Aaltomaa et al, 1993). We feel that most differences in the observed nuclear size and size variation among different publications are due to factors related to patient material and application of the morphometric method. In our experience, a strictly standardized and uniform measuring technique, with regular calibration of the computerized morphometric equipment with a micrometer slide, ensures reproducible results applicable to prognostication and classification on the basis of nuclear size (Kronqvist et al, 1997). The obvious advantages of nuclear morphometry also include inexpensive equipment, conceptually simple methods and reproducible results, which also facilitate the use of morphometry in routine pathology practice. Compared with subjective grading the morphometric method is, however, somewhat more time-consuming and demands specially trained personnel.

To sum up, the scope of the present study was to produce quantitative criteria for nuclear grading in breast cancer, and by this means to improve the consistency and the accuracy of the method. We have been able to introduce, for nuclear morphometric features in invasive ductal breast cancer, quantitative thresholds which can be developed for application in the traditional Bloom and Richardson grading as well as in other classification systems such as the Nottingham Prognostic Index. Our results suggest that the nuclear morphometric grading system can be used as the basis for treatment decisions in breast cancer and that mean shortest nuclear axis is the most significant morphometric prognosticator among the features tested. Obviously, this paper on morphometric nuclear grading is a preliminary one and other features of histological grading have to be similarly analysed for quantitative thresholds before we can speak of a true morphometric grading system. We are already in progress with corresponding studies on mitotic activity and tubular formation.

### ACKNOWLEDGEMENT

This work was supported by grants from the Cancer Foundation of South-West Finland.

### REFERENCES

Aaltomaa S, Lipponen P, Eskelinen M, Alhava E and Syrjänen K (1991) Nuclear morphometry and mitotic indexes as prognostic factors in breast cancer. *Eur J Surg* **157**: 319–324

Aaltomaa S, Lipponen P, Eskelinen M, Kosma VM, Marin S, Alhava E and Syrjänen K (1993) Comparison of classic and quantitative prognostic factors in hormone receptor-positive and hormone receptor-negative female breast cancer. *Am J Surg* **165**: 307–311

Baak JPA (1992) *Manual of Quantitative Pathology in Cancer Diagnosis and Prognosis*, p. 158. Springer-Verlag: Berlin

Baak JPA, Kurver PHJ, De Snoo-Niewlaat AJE, De Graaf S, Makkink B and Boon M (1982) Prognostic indicators in breast cancer – morphometric methods. *Histopathology* **6**: 327–339

Baak JP, Van Dop H, Kurver PH and Hermans J (1985) The value of morphometry to classic prognosticators in breast cancer. *Cancer* **56**: 374–382

Bloom HJG and Richardson WW (1957) Histologcal grading and prognosis in breast cancer. *Br J Cancer* **11**: 359–377

Collan Y, Kuopio T and Alanen K (1992) Scope and concepts of quantitative histopathology. *Acta Stereol* **11**: 3–23

Cutler SJ and Ederer F (1958) Maximum utilization of the life table method in analyzing survival. *J Chron Dis* **8**: 699–712

Dalton LW, Page DL and Dupont WD (1994) Histologic grading of breast carcinoma. A reproducibility study. *Cancer* **73**: 2765–2770

Davis BW, Gelber RD, Goldhisch A, Hartman WH, Locher GW, Reed R, Golouch R, Säve-Söderbergh J, Holloway L, Russel I and Rudenstam CM (1986). Prognostic significance of tumor grade in clinical trials of adjuvant therapy for breast cancer with axillary lymph node metastasis. *Cancer* **58**: 2662–2670

Early Breast Cancer Trialists Collaborative Group (1992). Systemic treatment of early breast cancer by hormonal, cytotoxic, or immune therapy. 133 randomised trials involving 31 000 recurrences and 24 000 deaths among 75 000 women. *Lancet* **339**: 71–85

Elston C-W and Ellis IO (1991) Pathological prognostic factors in breast cancer. I. The value of histological grade in breast cancer: experience from a large study with long-term follow-up. *Histopathology* **19**: 403–410

Frierson HF, Wolber RA, Berean KW, Franquemont DW, Gaffey MJ, Boyd JC and Wilbur DC (1995) Interobserver reproducibility of the Nottingham modification of the Bloom and Richardson histologic grading scheme for infiltrating ductal carcinoma. *Am J Clin Pathol* **103**: 195–198

Galen RS and Gambino SR (1975) Beyond normality. In *The Predicted Value and Efficiency of Medical Diagnosis*, pp. 49–51 and 115–116. John Wiley and Sons: New York

Henson DE, Ries L, Freedman LS and Carriaga M (1991) Relationship among outcome, stage of disease, and histologic grade for 22 616 cases of breast cancer: the basis for a prognostic index. *Cancer* **68**: 2142–2149

Kairisto V and Poola A (1995) Software for illustrative presentation of basic clinical characteristics of laboratory tests – GraphROC for Windows. *Scand J Clin Lab Invest* **55**: 43–60

Kronqvist P, Collan Y, Kuopio T and Kujari H (1995) Nuclear morphometry in breast cancer: the influence of sampling rules and freezing of samples. *Mod Path* **8**: 187–192

Kronqvist P, Kuopio T, Tamm Ü, Horvath C and Collan Y (1997) The reproducibility of nuclear morphometric measurements in invasive breast carcinoma. *Anal Cell Pathol* **15**: 47–59

Ladekarl M and Sörensen FB (1993). Quantitative histopathological variables in in-situ and invasive ductal and lobular carcinomas of the breast. *APMIS* **101**: 895–903

Larsson LG, Nystrom L, Wall S, Rutqvist L, Andersson I, Bjurstam N, Fagerberg G, Frisell J and Tabar L (1996) The Swedish randomized mammography screening trials: analysis of their effect on the breast cancer related excess mortality. *J Med Screen* **3**: 129–132

Moss SM, Ellman R, Coleman D and Chamberlain J (1994) Survival of patients with breast cancer diagnosed in the United Kingdom trial of early detection of breast cancer. *J Med Screen* **1**: 193–198

Pienta KJ and Coffey DS (1991) Correlation of nuclear morphometry with progression of breast cancer. *Cancer* **68**: 2012–2016

Robbins P, Pinder S, De Klerk N, Dawkins H, Harvey J, Sterrett G, Ellis I, and Elston C (1995) Histological grading of breast carcinomas: a study of interobserver agreement. *Hum Pathol* **26**: 873–879

© Cancer Research Campaign 1998

Robert NJ (1994) Adjuvant threrapy in breast cancer. *Obstet Gynecol Clin North Am* **21**: 693–707

Schöndorf H and Naujoks H (1985) Determining the nuclear area in normal breast epithelia and in the nuclei of mammary carcinomas. *J Cancer Res Clin Oncol* **109**: 241–244

Simpson J-F and Page DL (1994) Status of breast cancer prognostication based on histopathologic data. *Am J Clin Pathol* **102**: (Suppl. 1): 3–8

Stierer M, Rosen H and Weber R (1991) Nuclear pleomorphism, a strong prognostic factor in axillary node-negative small invasive breast cancer. *Breast Cancer Res Treat* **20**: 109–116

Styblo TM and Wood WC (1996) Adjuvant chemotherapy in the node-negative breast cancer patient. *Surg Clin North Am* **76**: 327–341

Tabar L, Fagerberg G, Duffy SW, Day NE, Gad A and Grontoft O (1992) Update of the Swedish two-county program of mammographic screening for breast cancer. *Radiol Clin North Am* **30**: 187–210

Uyterlinde AM, Baak JPA, Schipper NW, Peterse HJ, Meijer JWR, Vooys PG and Matze E (1991) Prognostic value of morphometry and DNA flow-cytometry features of invasive breast cancers detected by population screening: comparison with control group of hospital patients. *Int J Cancer* **48**: 173–181

Van der Linden HC, Baak JPA, Lindeman J, Hermans J and Meyer CJLM (1986) Morphometry and breast cancer. II. Characterisation of breast cancer cells with high malignant potential in patients with spread to lymph nodes: preliminary results. *J Clin Pathol* **39**: 603–609

Zweig MH and Campbell G (1993) Receiver-operating characteristic (ROC) plots: a fundamental evaluation tool in clinical medicine. *Clin Chem* **39**: 561–577

© Cancer Research Campaign 1998